# To be thin but not healthy - The body-image dilemma may affect health among female university students in China

**DOI:** 10.1371/journal.pone.0205282

**Published:** 2018-10-10

**Authors:** Lei Zhang, Haihong Qian, Hua Fu

**Affiliations:** 1 School of Public Health, Fudan University, Shanghai, China; 2 School of Basic Medical Sciences, Fudan University, Shanghai, China; Fordham University, UNITED STATES

## Abstract

An increasing number of young girls have attached great importance to their body-image in China. Body-image dissatisfaction has resulted in increased weight loss behavior. The aim of this study was to investigate the factors that were associated with underweight body-image in female college students. Self-administered questionnaires were completed by 2,023 young female participants from eight Chinese universities. In addition, 160 participants were involved in a qualitative study. The ideal body-image and the factors that influence weight were determined using descriptive and analytical statistics. We found that 1,484 out of 2,023 participants (73.36%) had taken action to lose weight in the past six months. Among these participants, 618 (30.55%, out of 2023) participants were already underweight, and 1,161 (57.39%, out of 2023) would like to be much thinner, which would lead to more underweight individuals. Moreover, non-scientific physical activity and diets were found to lead participants to the underweight subgroup. The participants’ Body Mass Index (BMI), peer advice and western culture influenced the problematic thin-ideal (ideal BMI < 18.5 was considered as the problematic thin-ideal) (*P*<0.05). Together, western influences leading to the “problematic thin-ideal” and “unhealthy weight-control behavior” have brought about an increased prevalence of desired underweight body-image among female college students in China, which might be harmful for their health.

## Introduction

In Asia, it was once believed that thinness was a sign of poverty and malnutrition [1, 2]. During the Chinese Tang Dynasty, obesity was even regarded as beauty. However, in the modern world, extreme ideals of thinness among women have become prevalent in both developed and developing countries, and there is a great dissatisfaction with body-image [3–9]. Body Mass Index (BMI), which divides weight in kilogram by height in meters square, is a commonly used international measure to evaluate human body fat and health. A BMI <18.5 is considered underweight, BMI from 18.5 to 25.0 is considered normal weight, BMI from 25.0 to 28.0 is considered overweight, and BMI >28 is considered obese [10, 11]. The knowledge about BMI will help to authenticate the weight and help to decide the behavior to keep fit body-image. Numerous studies have shown that, despite being normal or underweight, many women perceive themselves as being overweight [12, 13]. It has been reported that 28% of college students in the United States who were underweight or normal weight were still trying to lose weight and, among US adults, 30% of males and 42% of females who perceived themselves as not overweight still practiced weight control behavior [14]. This tendency also has emerged in Asia. Some researchers have suggested that young women in Asian countries have adopted ideal body types thinner than their actual figures. Currently, in China, underweight has become synonymous with beauty, causing image issues among female university students. Advertisements for weight-loss products are prevalent in China, and there are an abundance of weight-loss products and programs in the marketplace [15, 16]. Non-overweight individuals may also feel pressure to achieve weight-loss goals by these weight-loss products and programs. Women may be more likely than men to be influenced by appearance pressures and experience more body dissatisfaction [17].

Individuals who consider a BMI <18.5 to be ideal were believed to have the thin-ideal. The female thin-ideal has become a potent contributor to the high levels of disordered eating and body-image disturbance. We consider this thin-ideal to be problematic, since BMI <18.5 belong to underweight and will jeopardize health in the long term, which may reduce sex hormones and bone mineral density and lead to anemia, low blood pressure, fatigue, discomfort and eating disorders, including anorexia nervosa, bulimia nervosa, and binge eating disorders. These disorders are associated with a wide range of damaging consequences for physical and mental health [18–22]. In addition, being underweight increases cardiovascular risks [23]. Some studies also have indicated that underweight can lead to infertility and preterm birth [24–27], which is particularly concerning for young women of childbearing age.

Nevertheless, there have been few reports on unhealthy weight-control behavior, on the proportion of non-overweight individuals among university students, or on what make these behaviors so prevalent in China. We speculated whether the unhealthy weight-control behavior was prevalent in the non-overweight individuals among university students. The aim of this research was to assess the prevalence of weight-loss behaviors among non-overweight individuals and the sociodemographic and behavioral factors that may affect these behaviors in female university students in China to promote health education campaigns and improve the health of young Chinese women.

## Materials and methods

### Setting and participants

The study consists of the quantitative and qualitative portions. In quantitative portions we used self-administered questionnaire, and in qualitative portions we used focus group discussions. In the quantitative research, a teacher from each university was asked to manage a survey in his/her university. In the qualitative research, teachers with certificate of professional psychological counselor conducted the discussion. The cross-sectional survey was performed from November 2016 to January 2017. The survey was administered at eight universities in different Chinese districts. Four of the districts are in municipalities that are directly under the administration of the Central Government, and the remaining four are in other urban districts. We used a systematic sampling method to ensure a representative sample from the universities in China with regard to region, school size and school type. We designated the eight universities as A-H University to protect the interviewees' privacy.

Participants were recruited among female students using a multistage stratified sampling approach with three steps. For the first step, we drew samples according to major, including literature, science, engineering, medicine, law, economics and management. Second, within every stratum, samples were further stratified based on three education levels, i.e., undergraduates, master’s candidates and Ph.D. candidates. In the steps above we also took the proportions of the population size of every stratum into consideration while conducting the probabilistic sampling. In the third step, individuals within various strata were drawn randomly according to their student ID. A total of 2,200 questionnaires were distributed (275 copies per university) and 2,050 were returned. After excluding 27 questionnaires with incomplete answers, 2,023 were determined as valid and introduced into the study. Before data collection, a teacher from each university was asked to manage a survey in his/her university, guide the participating students to administer the questionnaire, and collect the questionnaires from the students.

### Ethical approval

Approval was granted by the ethics committee of the School of Public Health of Fudan University, China (IRB00002408&FWA00002399). Informed verbal consent was given by all participants. The right to withdraw and to autonomy was explained to the respondents.

### Anthropometric assessment

The weight and height of the participants were measured by the universities’ health units using a standardized method. Weight was measured without shoes and heavy clothes and was recorded to the nearest 250 g. Height was measured without shoes and was recorded to the nearest 0.5 cm.

### Body mass index (BMI)

Body Mass Index (BMI) divides weight in kilograms by height in meters squared and is a commonly used international measure to evaluate human body fat and health. According to the world and Chinese standards, BMI <18.5 is considered underweight [10, 11], BMI from 18.5 to 25.0 is considered normal weight, BMI from 25.0 to 28.0 is considered overweight, and BMI >28 is considered obese.

Actual BMI = weight (kg) (measured weight) / height^2^ (m)

Ideal BMI = weight (kg) (ideal weight response from the self-administered questionnaire) / height^2^ (m)

### The problematic thin-ideal

Participants whose ideal BMI was <18.5 were considered to have the problematic thin-ideal, and those with ideal BMI between 18.5 and 25 were considered to have a healthy ideal.

### Extreme methods in weight-control behaviors

We explained “extreme weight-control behaviors” and provided three examples to participants in the self-administered questionnaire and the interview, including gastrectomy, vomiting after meals, and liposuction. Gastrectomy involves the removal of the left side of the stomach by surgery, and liposuction is a surgical procedure that uses a suction technique to remove fat from specific areas of the body, including the abdomen, hips, thighs, buttocks, arms and neck. We also asked participants to supplement other potential “extreme weight-control behaviors”, and in the interview and questionnaire some students supplemented “acupuncture”. Therefore, a total of four kinds of “extreme weight-control behaviors” were included in the final analysis.

### Self-administered questionnaire

The self-administered questionnaire is part of “Indicators System of Health Literacy Assessment for College Student in China” and the questionnaire was measured as an individual-level variable. The self-administered questionnaire was based on the Chinese population. A convenience sample of 2,023 participants from eight universities was investigated. The results showed good construct validity and content validity. Each participant was given a questionnaire that covered the following topics: demographic data, possible factors that affected body-image, possible influencing factors that made the participant decide to change their weight, and the mechanism they used to try to lose weight.

In the self-administered questionnaire, participants were asked “Do you know about BMI”, and there were three choices as follows: (A) I know about BMI. I know how to calculate it and know the BMI standard; (B) I have heard about BMI, but I don’t know how to calculate it or the standard BMI; (C) I don’t know anything about BMI. The option was a single choice of three levels of frequency.

### Qualitative study

After the self-administered questionnaire, we performed a qualitative study. In each university, 20 participants, divided into 2 groups with 10 participants each, were invited to take part in an interview to gather in-depth information. A total of 160 participants took part in the study. All of these participants had already completed the self-administered questionnaire. The 10 participants in each group had an hour for discussion. The discussions were led by a member of our research group. We used the question formats of an in-depth interview, semistructured interview and structured interview. Before the interview, we discussed the frame of the interview. Using the verbal probing method, the interviewer probes further to understand the survey responses by following up with a series of related questions. The frame is as follows:

1. Are you satisfied with your body-image? What is your ideal body-image? What is your standard of fitness?
2. Why do you want to change your weight? Whose advice made you think you were not thin enough?
3. Have you lost weight in the last 6 months? If you have, please tell us how you control your weight.
4. If you do exercise, do you have an instructor? What kind of sports do you do? And how often and how long each time?
5. If you diet, which kind of diet do you choose? Dukan diet, Copenhagen diet, JungDaYeon diet, or other kinds of weight-loss diets. In addition, where did you find this diet? Or do you just reduce the meal portions, reduce/cut out some types of food, or skip one/two meal(s) per day. Or do you have other ways to diet?
6. Do you try other mechanism, such as vomiting after eating or liposuction? Is it helpful?
7. Whose advice did you take to make you decide to lose weight? What did they say?

### Statistical analysis

The data were entered and managed with EpiData 3.1 (The EpiData Association, Odense, Denmark). The analyses were performed with SPSS (Statistical Package for the Social Sciences) version 20.0 (IBM, Chicago, IL, USA). A frequency analysis and Pearson’s chi-square test were used to examine the association between demographic variables (age, education level, home location, mother’s/father’s education level, school location, monthly allowance, and self-reported BMI). Moreover, a multiple logistic regression analysis was applied to evaluate the association between body-image and these demographic variables. Odds ratios (ORs), 95% confidence intervals (CIs), and *p* values are presented for the multivariable models. Actual BMI and ideal BMI are shown as mean, standard deviation (SD), and range. The multiple logistic regression analysis was used to evaluate the possible factors impacting body-image dissatisfaction. Pearson’s chi-square test was used to assess the behaviors for weight control among participants according to their actual BMI. Level of significance was set to *p* < 0.05.

## Results

### Demographic characteristics of the BMI subgroups

The demographic characteristics (age, education level, mother’s/father’s education level, monthly allowance, and self-reported BMI) of the participants and their BMI subgroups were obtained from questionnaire and shown in Table 1. According to their actual BMI, the participants were classified into three groups as follows: underweight (539, 26.62%), normal weight (1,443, 71.11%), overweight / obese (41, 2.27%). Pearson’s chi-square test was used to analyze the correlation between mother’s education level and BMI subgroup, and a significant difference was found (X^2^ = 45.05, *P* < 0.05); however, the correlation between father’s education level and BMI subgroup was not observed (X^2^ = 17.85, *P* = 0.09). In addition, 651 (82.95%) participants in the normal subgroup were knowledgeable about BMI, which was much more than that in the underweight subgroup (131 participants, 16.69%) and overweight subgroup (3 participants, 0.38%). Pearson’s chi-square test also confirmed the correlation between BMI knowledge and the participants’ subgroup with statistical significance (*P* < 0.05). Therefore, the mothers’ education level and self-definition of BMI affected the BMI subgroup.

**Table 1:** Correlation between demographic characteristics and the BMI subgroups.

Variables	Total	Underweight	Normal	Overweight/Obese		X^2^	*P*-value
	n = 2023	n = 539(%)	n = 1443(%)		n = 41(%)		
Age range (y)						8.90	0.064
<20	416	101(24.28)	308(74.04)		7(1.68)		
20–25	1427	402(36.77)	993(38.67)		32(60)		
>25	180	36(6.67)	142(9.84)		2(0)		
Education level						1.70	0.426
Undergraduate	1499	399(26.62)	1066(71.11)		34(2.27)		
Postgraduate	524	140(29.58)	377(68.59)		7(1.83)		
Mother’s Education level						45.05	**<0.001**
Primary school	286	89(31.12)	189(66.08)		8(2.80)		
Secondary school	978	312(31.90)	639(65.34)		27(2.76)		
College	759	138(18.18)	615(81.03)		6(0.79)		
Father’s Education level						17.85	0.09
Primary school	184	85(46.20)	91(49.46)		8(4.34)		
Secondary school	1020	298(29.22)	697(68.33)		25(2.45)		
College	819	156(19.05)	655(79.98)		8(0.98)		
Monthly allowance (in RMB)						12.79	0.12
<1,000	301	84(27.91)	210(69.77)		7(2.33)		
1,000–2,000	1131	278(24.58)	823(72.77)		30(2.65)		
>2,000	591	177(29.95)	410(69.37)		4(0.68)		
BMI knowledge						129.03	**<0.001**
Knowledgeable	785	131(16.69)	651(82.95)		3(0.38)		
Little knowledge	354	159(44.92)	181(51.12)		14(3.95)		
No knowledge	884	249(28.16)	611(69.12)		24(2.71)		

### Factors associated with the underweight BMI subgroup

The factors related to the underweight BMI subgroup were obtained from questionnaire, and multinomial logistic regression analysis was applied (Table 2). The model outcome was the underweight BMI subgroup with the normal BMI subgroup as the reference group. A low level of BMI knowledge (little: OR = 1.59, 95% CI = 1.32–2.11; nothing: OR = 1.97, 95% CI = 1.46–2.78) was significantly associated with the underweight BMI subgroup. Those with a low level of BMI knowledge were more likely to be underweight (*P* < 0.05).

**Table 2:** Influencing factors associated with the underweight BMI subgroup.

Variables	Underweight	
	OR	95% CI
Age (y)	1.23	1.11–1.42
Education level		
Undergraduate	1	NA
Postgraduate	0.93	0.76–1.17
Mother’s Education level		
Primary school	1	NA
Secondary school	1.34	0.99–1.67
College	0.87	0.68–1.02
Father’s Education level		
Primary school	1	NA
Secondary school	1.08	0.91–1.27
College	0.96	0.87–1.05
Monthly allowance (in RMB)		
<1,000	1	NA
1,000–2,000	0.98	0.28–2.03
>2,000	1.01	0.13–3.11
BMI knowledge		
Knowledgeable	1	NA
Little knowledge	1.59*	1.32–2.11
No knowledge	1.97***	1.46–2.78
Physical activity	1.34	0.32–2.75
Guide by a book or video	1.78*	1.33–2.01
Guide by a fitness coach	0.88	0.17–2.06
Diet	2.15**	1.89–2.67
Guide by a book or the internet	2.84**	2.23–3.05
Guide by a dietitian	0.49	0.23–0.88
Diet pills	2.35**	1.76–2.68
Extreme methods	3.11**	2.12–3.85

*, *P* < 0.05

**, *P* < 0.01

***, *P* < 0.001

In addition, we evaluated the participants’ behavior in the last 6 months in the underweight BMI subgroup. First, we grouped weight-control behavior into the following categories: physical activity, diet, diet pills and extreme methods (for example: vomiting after meals and liposuction). Then, we grouped different kinds of physical activity into the following three subgroups: (1) Reducing meal portions or reducing/eliminating certain types of food without a guide; (2) Using a guide from the internet or a book, such as Keep (APP) or JungDaYeon body building exercise; and (3) Guided by a fitness coach. Finally, we grouped diet into the following three subgroups: (1) Participating in sports such as jogging or swimming without an instructor; (2) Using instructions from a book or video/App, such as Dukan diet; and (3) Guided by a dietitian. Physical activity guided only by a book or video (OR = 1.78, 95% CI = 1.33–2.01), diet behavior (OR = 2.15, 95% CI = 1.89–2.67), diet guided only by a book or the internet (OR = 2.84, 95% CI = 2.23–3.05), taking a diet pill (OR = 2.35, 95% CI = 1.76–2.68), and using extreme methods (OR = 3.11, 95% CI = 2.12–3.85) were significantly associated with underweight BMI. Those who exhibited weight loss behaviors were more likely to be underweight (*P* < 0.05). In addition, we also observed that over 90% of underweight individuals tried to lose weight without the help of a professional.

In the qualitative study, we gathered in-depth information in the discussion group about how participants tried to control their weight. A total of 113 of 160 participants said they had tried to control their weight, and over 90% of underweight individuals had tried to lose weight without the help of a professional. The detailed behaviors of these individuals are shown in Table 3. A total of 65 interviewees (40.6%) jogged, which was the most popular exercise method. A total of 73 interviewees (45.63%) reduced meal portions. In the interview, a 19-year-old participant from C University told us, “I just want to be thinner. I know more physical activity and less food will be helpful, and these two methods are the most accessible ways to control weight. I do not think it is necessary to go for a professional consultant and rely on his/her advice in losing weight. In addition, I have already lost 5 kg weight. Now I weigh 50 kg and I am 165 cm tall.” A 24-year-old participant from F University said, “I searched for weight-loss diets on the internet. I have tried the Dukan diet for almost one year and have already lost 15 kg weight. I plan to continue with it. I do not think I need a guide. My method is useful.” We found most of the interviewers (96.25%) tried to lose weight in their own ways, which may have led to becoming underweight.

**Table 3:** “Weight-control behaviors” in the qualitative study.

Variable	Underweight	
	n = 160	%
Physical Activity Behaviors		
Jogging	65	40.63%
Body building exercise	27	16.88%
Yoga	19	11.88%
Swimming	13	8.13%
Dance	7	4.38%
Physical activity-guide App/Video		
Keep (App)	15	9.38%
JungDaYeon body building exercise	13	8.13%
Iphone health (App)	9	5.63%
Nike+ (App)	5	3.13%
Other exercise App	4	2.5%
Diet		
Reduce meal portions	73	45.63%
Reduce/eliminate some types of food	49	30.63%
Skip one/two meal(s) per day	23	14.38%
Diet program from book/internet		
Dukan diet	2	1.25%
Copenhagen diet	3	1.88%
JungDaYeon diet	3	1.88%
Other weight-loss diet	7	4.38%
Extreme methods		
Acupuncture	2	1.25%
Vomiting after meal	3	1.88%
Liposuction	1	0.63%

### Association between the desire to change weight and BMI subgroup

The ideal BMI was calculated using the ideal weight and actual height that participants reported in the self-administered questionnaire. Table 4 shows the actual BMI and ideal BMI of the participants. A total of 1,292 participants (63.87%) were dissatisfied with their actual BMI. We assessed their desire to change weight and found that 1,164 (57.54%) participants wanted to lose weight, 498 (24.62%) participants wanted to keep their actual weight, and only 361 (17.84%) participants wanted to gain weight. The mean ideal BMI among the participants was 19.395 ± 1.755, and the mean ideal BMI of the underweight subgroup was 17.995 ± 0.862. A 19-year-old interviewer from B University, who’s BMI was 17.45 told us, “I am 165 cm tall and weigh 47.5 kg, but I still think I am fat and need to lose weight. I want to have an A4 waist; it is my target.” An “A4 waist” means a waist that can be covered by an A4 sheet of paper, indicating a waist width under 21 cm.

**Table 4:** Association between the desire to change weight and BMI subgroups.

Variables		Underweight	Normal	Overweight/Obese	Total	X^2^	*P*-value
		n = 539(%)	n = 1443(%)	n = 41(%)	n = 2023(%)		
Body-image satisfaction						17.72	**<0.001**
Satisfied		198(36.73)	531(36.80)	2(10)	731(36.13)		
Dissatisfied		341(63.27)	912(63.20)	39(90)	1292(63.87)		
Weight change						660.88	**<0.001**
Be thinner		107(19.85)	1016(70.41)	41(100)	1164(57.54)		
No change		161(29.87)	337(23.35)	0(0)	498(24.62)		
Be fatter		271(50.28)	90(6.23)	0(0)	361(17.84)		
IdealBMI	Mean±SD	17.995±0.862	19.730±1.252	25.995±2.301	19.395±1.705		
	Range	15.57–20.70	16.59–24.14	22.49–33.06	15.57–33.06		
Problematic thin-ideal		413(76.62)	205(14.21)	0(0)	618(30.55)	725.43	**<0.001**
Healthy ideal		126(23.38)	1238(85.79)	20(47.48)	1384(68.41)		

We assumed that 413 participants in the underweight group and 205 participants in the normal group who wished to have a BMI under 18.5, had the problematic thin-ideal. Similarly, we assumed that the 1,364 (67.42%) participants who wanted a BMI approximately 18.5 to 25 had a healthy ideal. Pearson’s chi-square test was used to analyze the data of the ideals by subgroup. Significant differences were found (*P* < 0.05) between the two subgroups, suggesting that the underweight subgroup may be more likely to have problematic thin-ideal.

### Factors influencing the “problematic thin-ideal”

A multinomial logistic regression analysis was used to determine the factors influencing the “problematic thin-ideal” obtained from questionnaire. The model outcome in this analysis was the “problematic thin-ideal”, using the “healthy ideal” group as the reference (Table 5). Following female friends’ advice to lose weight (OR = 4.26, 95% CI = 2.45–7.44), following male friends’ advice to lose weight (OR = 3.31, 95% CI = 2.01–4.21), focusing on fashion (always/often, OR = 2.11/1.91, 95% CI = 1.78–2.79/1.38–2.57, respectively), watching entertainment programs (always/often, OR = 1.68/1.05, 95% CI = 0.99–2.34/0.87–1.39, respectively), and losing weight for appearance (OR = 4.83, 95% CI = 2.64–8.84) were found to be significant. In an interview, an 18-year-old participant from A University said, “I am 165 cm tall and weigh 50 kg. My parents think I am too thin, but my roommates do not think so. They suggest that I would be more beautiful if I can lose 2.5 kg. I agree with my roommates, so I decided to lose weight.” Another 22-year-old participant from H University said, “I am dissatisfied with my weight only because my boyfriend wants me to lose weight. I do not think others’ attitudes will change my thoughts, except those of my boyfriend.” Those who took their friends’ advice, focused on fashion information and watched entertainment programs were more likely to have the “problematic thin-ideal” (*P* < 0.05). Additionally, most of the participants (76.15%) worked toward an underweight BMI for appearance but not for health.

**Table 5:** Factors influencing the “problematic thin-ideal”.

Variables	“Problematic thin-ideal”	
	OR	95% CI
Taking a female friend’s advice to lose weight	4.26***	2.45–7.44
Taking a male friend’s advice to lose weight	3.31***	2.01–4.21
Taking a family member’s advice to lose weight	1.21	0.98–1.45
Focus on fashion information		
Always (over 10 hours every week)	2.11**	1.78–2.79
Often (2–10 hours every week)	1.91**	1.38–2.57
Never (less than 1 hour every week)	1	NA
Watch entertainment programs		
Always (over 10 hours every week)	1.68*	0.99–2.34
Often (2–10 hours every week)	1.05*	0.87–1.39
Never (less than 1 hour every week)	1	NA
For health	1.33	1.01–1.78
For appearance	4.83***	2.64–8.84

*, *P* < 0.05

**, *P* < 0.01

***, *P* < 0.001

In the interview, many interviewers also provided some details when talking about the effect of culture. A 20-year-old participant from F University said, “Currently, American culture is the most popular; Japanese and Korean culture are also welcome among young people. In these cultures, almost all of the female pop stars are thin. Therefore, to be beautiful, the first step is to be thin.”

## Discussion

Our cross-sectional survey described a phenomenon in which 372 out of 539 participants (69.02%) in the underweight subgroup had tried to lose weight in the 6 months prior to the survey. Almost all of these 372 participants chose to perform physical activity and go on a diet. However, less than 10% of the participants had a plan or looked for guidance, and almost no one sought professional guidance. Most of the participants tried to lose weight by unhealthy means. In the study, we found that 361 participants (17.84% of the 2023 participants) wanted to gain weight. Among these 361 participants, 271 out of them belonged to the underweight subgroup. Nevertheless, 145 out of the 271 underweight participants still wanted to retain the underweight BMI (less than 18.5), suggesting that they bear the “problematic thin-ideal.” Of the 2,023 participants, 618 (30.55%) participants had an ideal BMI lower than 18.5, suggesting that they had a similar problematic thin-ideal. In 2008, Ahern et al. found that females rated a significant number of underweight female bodies as “normal-weight” and that normal-weight female bodies were consistently labeled “overweight.” By using weight loss mechanisms, more and more young girls might become underweight. “Unhealthy weight-control behavior” leading to an underweight body-image was prevalent among female university students, which may impede the health of young women in China.

In Asia, it was once believed that thinness was a sign of poverty and malnutrition [1, 2]. However, the pursuit of a slim body has become widespread among females all over the world [7–9]. In our research, 26.64% of the participants were underweight. Furthermore, 63.87% of them were dissatisfied with their actual weight, 57.74% wanted to lose weight, and 30.55% showed a problematic thin ideal. Thus, it seems that the prevalence of an extremely thin-body ideal among young Chinese girls was similar to or even more serious than what has been reported among girls from other countries [28,29]. Although Western and non-Western cultures are distinct, body-image dissatisfaction seems to be similar [30–34], with a growing emphasis on an ultra-thin ideal body. Additionally, we found that 43.7% of participants knew nothing about BMI, making it less likely that they had a normal BMI. Thus, the thin-body ideal poses a potentially serious threat to the health of young people.

Unhealthy weight control behavior and being thin is now prevalent among female students in Chinese universities, and it has even become fashionable. Some studies [34, 35] suggest that mothers and sisters influence the motivation for underweight and body dissatisfaction. In our research, we found that peer suggestion was the main factor affecting a participant’s judgment of her own body-image. Peer advice also influenced the decision to engage in unhealthy weight-loss behavior, whereas advice from family members was commonly ignored. As indicated in both the surveys and interviews, females pay more attention to their peers' judgment of their appearance, which influences their body-image [36]; this influence is both on the behavior and the view of body-image.

Currently, Asian culture already agrees on “the thinner the better” for female stars, and most advertisements and programs regard thinness as a symbol of beauty [37]. In our study, we also found that the ideal body-image is strongly correlated with popular culture among Chinese young women. The source of sociocultural influence most heavily criticized for promoting body dissatisfaction and body change behaviors is culture, with its relentless portrayal of thin female bodies and emphasis on diet and weight control. It has been suggested that in non-Western countries, exposure to Western media leads to the adoption of Western beauty ideals, which in time overrides traditional or previously held body type ideals. As Shih and Kubo demonstrated in 2002, Japan and Taiwan have been heavily exposed to Western culture, and both populations have relatively similar body sizes. Therefore, it is uncertain if Chinese attitudes and values regarding body-image can withstand the influence of western culture. In our study, although the interviewers said Asian culture had a slightly stronger influence, we also found that, with the international cultural exchange, Asian culture absorbs western values and exhibits more in common with western culture. These factors provide support for Jung and Forbes’ argument in 2007 that the relationship between body dissatisfaction and culture is complex and multifactorial and highlights the importance of interpreting the influence of culture within the context of the predominant culture.

In addition, both judgment from society and the media drive female university students to believe in a “fit and healthy” body. An 18-year-old student from H University said, “Now it is believed that less than 50 kg is fit.” Indeed, comparisons to the bodies idealized in the media and in social contexts have been found to be associated with body-image dissatisfaction, especially in young females [38–42]. The objectification theory [43] contends that females are constantly objectified and that body-image is often used in the assessment of their personal worth by others. Objectification theory posits that girls and women are typically acculturated to internalize an observer's perspective as a primary view of their physical selves. This perspective on self can lead to habitual body monitoring, which, in turn, can increase women's opportunities for shame and anxiety, reduce opportunities for peak motivational states, and diminish awareness of internal bodily states. Accumulations of such experiences may help account for an array of mental health risks that disproportionately affect women, i.e., unipolar depression, sexual dysfunction, and eating disorders. Objectification theory also illuminates why changes in these mental health risks appear to occur in step with life-course changes in the female body. Thus, females internalize that their self-worth is based on the views of others, and this leads to their own subconscious social judgment on their body-image. Through our interviews, the influence of the internet was also discovered. Over half of the students mentioned popular internet expressions, such as “A4 waist”, “the belly button challenge” (which involves touching your navel by reaching behind your back and around your waist), “balancing coins on collarbones,” and “firm abs”. All these phrases allude to an aim for weight loss rather than keeping health.

There are still some limitations in our study. Our cross-sectional study suggests that a “problematic thin-ideal” and “unhealthy weight-control behavior” resulting in a low to modest BMI have already emerged as a significant issue among female students in Chinese universities. Additionally, it is distressing that this desire is not motivated by body weight but by a negative opinion toward one’s own body-image and the desire to fit social norms of appearance [44]. We focused on the undesirable tendency in the group of female university students but did not assess the students diagnosed with eating disorders. While diagnosable eating disorders have a large effect on body-image, it is difficult for us to distinguish disorders for these participants because diagnosable eating disorders are usually caused by mental problems and college students are often private about these conditions. Therefore, it is unlikely for us to obtain this information in a self-administered questionnaire. To comprehensively understand the harm of this phenomenon, it would be better to perform a longitudinal study in other young females in addition to college students. This information may help to reduce this serious threat to the health of young Chinese females.

## Conclusion

Our data indicated that a “problematic thin-ideal” and “unhealthy weight-control behavior” that led to an underweight body-image was prevalent among female students in Chinese universities. Culture is the main source of the common thought that a thinner body makes a person more beautiful, and the “problematic thin-ideal” has become a potent contributor to body-image dissatisfaction among the female population. The high frequencies of “unhealthy weight-control behavior”, including eating disorders and over-exercise, has led to an increase in the prevalence of underweight females and could be harmful to the overall health of young women [45]. Helping young females keep away from the “problematic thin-ideal” as well as “unhealthy weight-control behavior” and teaching them to control weight according to a scientific standard is essential and needs more public attention.

## Supporting information

S1 Table Original data of self-administered questionnaire. The original data obtained from self-administered questionnaire are shown in this table.
(XLSX)

S2 Table Original data of qualitative study. The original data obtained from qualitative study are shown in this table.
(XLSX)
